# Long survival in a pancreatic carcinoma patient with multi-organ toxicities after sintilimab treatment: A case report

**DOI:** 10.3389/fphar.2023.1121122

**Published:** 2023-01-20

**Authors:** Chen-Xu Ni, Yu Zhao, Hong Qian, Hui Fu, Yu-Ying Yan, Yu-Shuang Qiu, Can-Can Zhou, Fang Huang, Fu-Ming Shen, Dong-Jie Li, Qing Xu

**Affiliations:** ^1^ Department of Pharmacy, Shanghai Tenth People’s Hospital, Tongji University School of Medicine, Shanghai, China; ^2^ Department of Oncology, Shanghai Tenth People’s Hospital, Tongji University School of Medicine, Shanghai, China; ^3^ Department of Pharmacy, Nanjing Medical University, Nanjing, China

**Keywords:** PD-1, pancreatic carcinoma, immune-related adverse events, long survival, case report

## Abstract

Pancreatic carcinoma is the leading cause of death among digestive malignancies in China. In particular, there is no breakthrough in prolonging the survival of pancreatic cancer patients with chemical and targeted therapies. Tumor immunotherapy brings opportunities and progress for the treatment of pancreatic cancer. Sintilimab is an innovative PD-1 inhibitor which was reported certain clinical benefits in multi-line treatments of advanced pancreatic cancer with gemcitabine. The combination therapy of PD-1 with gemcitabine plus high-intensity focused ultrasound (HIFU) in pancreatic cancer has not been reported. Here we report a case of a Chinese old patient diagnosed with metastatic pancreatic cancer. Two months after sintilimab treatment, the patient occurred severe immune colitis. The patient was diagnosed with immune ureteritis after 8 months of treatment. The immue-related adverse events (irAEs) refined after timely recognition and correct intervention by the clinician and clinical pharmacist. After first-line treatment of sintilimab plus gemcitabine combined with pancreatic HIFU, the patient achieved a remarkable benefit of 11-month progression-free survival (PFS) and 20-month overall survival (OS). The first-line treatment of sintilimab plus gemcitabine combined with HIFU demonstrates a potential therapeutic effect on metastatic pancreatic carcinoma with tolerable adverse reactions.

## 1 Introduction

Pancreatic carcinoma is a malignant tumor of digestive system with a very high degree of malignancy and poor prognosis. In China, the incidence and mortality of pancreatic cancer are annually increasing, and the mortality rate is close to the incidence (Cao et al., 2021). Pancreatic cancer is characterized by strong occult, aggression, easy to metastasize, and tolerant to chemoradiotherapy. The 5-year survival rate of pancreatic cancer is very low (less than 8%), and the median survival time is only 6 months (McGuigan et al., 2018). The main methods of treatment for pancreatic cancer include surgery, radiotherapy, chemotherapy and interventional therapy. Many clinical studies have shown the advantage of HIFU in pain reduction, extension of survival time, improvement of performance status and the great safety in advanced pancreatic patients, which confirmed it as a promising modality for palliative therapy (Xiaoping and Leizhen, 2013; Zhou, 2014; Marinova et al., 2016). It has been approved by the China Food and Drug Administration (CFDA) for the treatment of metastatic pancreatic cancer.

Therapy that targets programmed death-1 or programmed death-ligand 1 (PD-1/PD-L1), which are known as immune checkpoints, has been rapidly developing as oncotherapy for various carcinomas recently. PD-1 inhibitors restore endogenous antitumor T Cell responses by blocking the interaction of PD-1 with its ligand PD-L1 (Han et al., 2020). The development of tumor immunotherapy has been brought opportunities and progress to the treatment of pancreatic cancer (Mucileanu et al., 2021). Although PD-1 inhibitors have produced impressive results on varied cancers, they also caused a series of immune-related adverse events (irAEs), which often involve the skin, intestine, liver, lung, endocrine and other target organs (Darnell et al., 2020). Given the immune-mediated activity of PD-1 inhibitors, there has been speculation regarding the prognostic value of irAEs. Some experts consider irAEs to be a projection of the overall immune response to PD-1 inhibitors, and hence irAEs could be used to gauge the overall tumor response or drug efficacy. However, the value of irAEs as a predictive marker for better patient survival is still debated.

An innovative PD-1 inhibitor Sintilimab is a human immunoglobulin G4 (IgG4) monoclonal antibody that can specifically bind PD-1 molecules on the surface of T Cells, thereby blocking the PD-1/PD-L1 pathway leading to tumor immune tolerance. In December 2018, Sintilimab was approved by Chinese National Medical Products Administration for the treatment of relapsed or refractory classical Hodgkin’s lymphoma after at least second-line systemic chemotherapy. In November 2019, Sintilimab became the first PD-1 inhibitor in China to be listed in the National Medical Insurance Directory. In 2021, sintilimab was added as an indication in combination with other drugs for the first-line treatment of unresectable advanced or recurrent squamous non-small cell lung cancer, and unresectable or metastatic hepatocellular carcinoma. More than two dozen clinical studies (more than 10 of which are registered clinical trials) are currently underway worldwide to evaluate the antitumor effects of sintilimab in various solid and hematologic tumors (Guo et al., 2022; Hao et al., 2022; Wang et al., 2022; Zeng et al., 2022).

**FIGURE 1:**
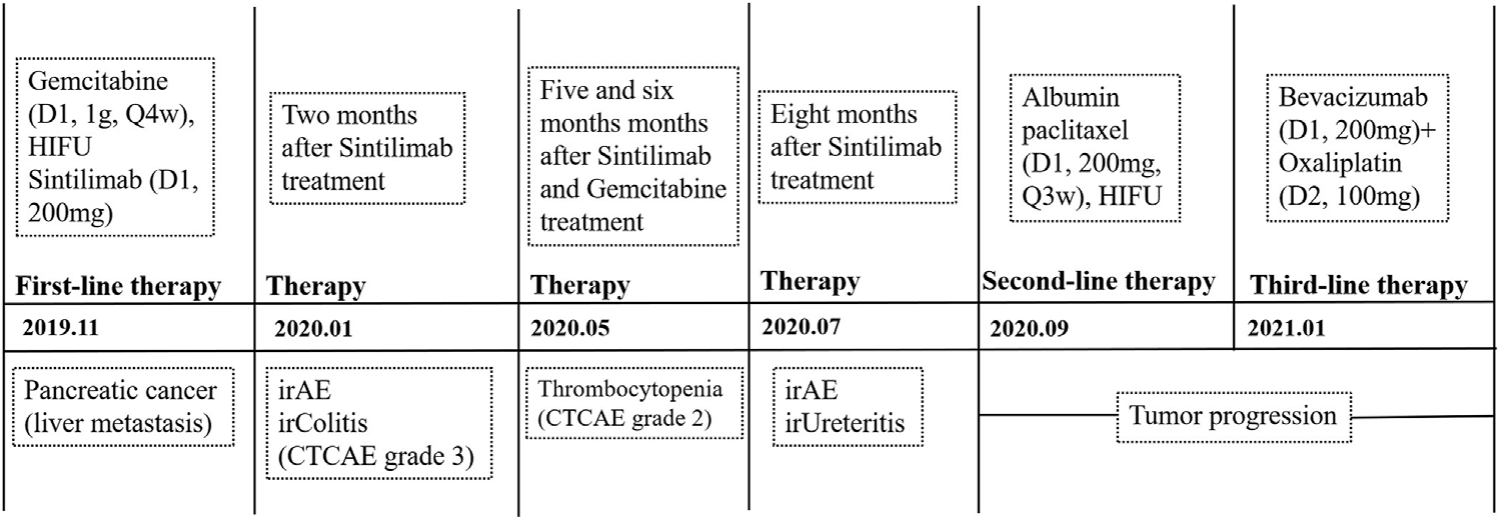
Schematic timeline of PD-1 treatment and its immune-related adverse events.

The case that we reported a Chinese old patient diagnosed with metastatic pancreatic cancer. Two months after Sintilimab treatment, the patient occurred severe immune colitis. The patient was diagnosed with immune ureteritis after 8 months of treatment. The irAEs improved after timely recognition and correct intervention by the clinician and clinical pharmacist. After the first-line treatment of Sintilimab plus gemcitabine combined with pancreatic HIFU, the patient achieved a remarkable benefit of 11-month PFS and 20-month OS (Figure 1).

## 2 Case presentation

**FIGURE 2:**
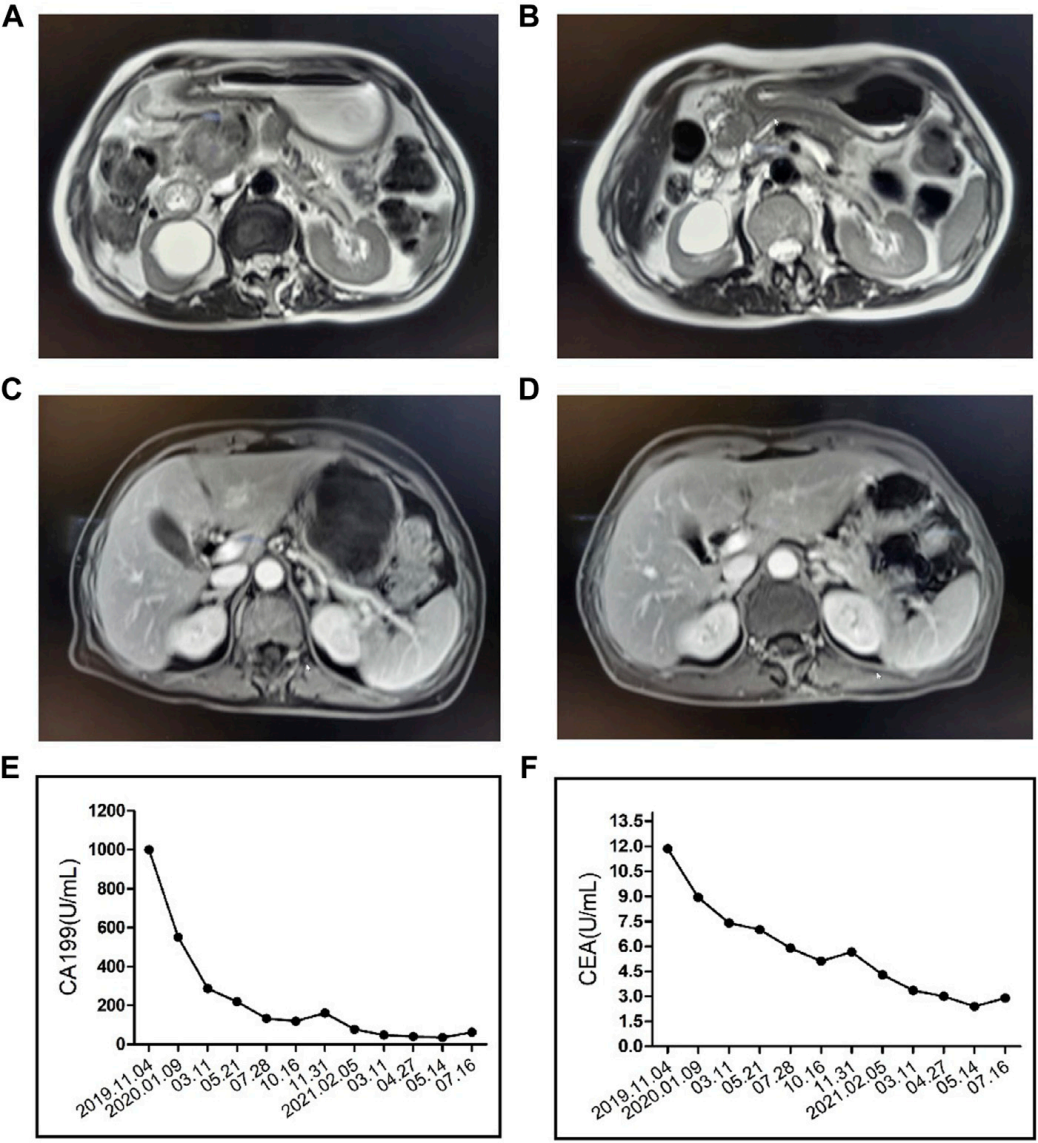
Contrast-enhanced Magnetic Resonance Imaging (MRI) of the upper abdomen showed the patient’s pancreatic carcinoma before **(A)** and after **(B)** the treatment of PD-1, HIFU and Gemcitabine. MRI showed the patient’s liver metastasis before **(C)** and after **(D)** the treatment. CA199 **(E)** and carcinoembryonic antigen (CEA) **(F)** index of the patient.

**FIGURE 3:**
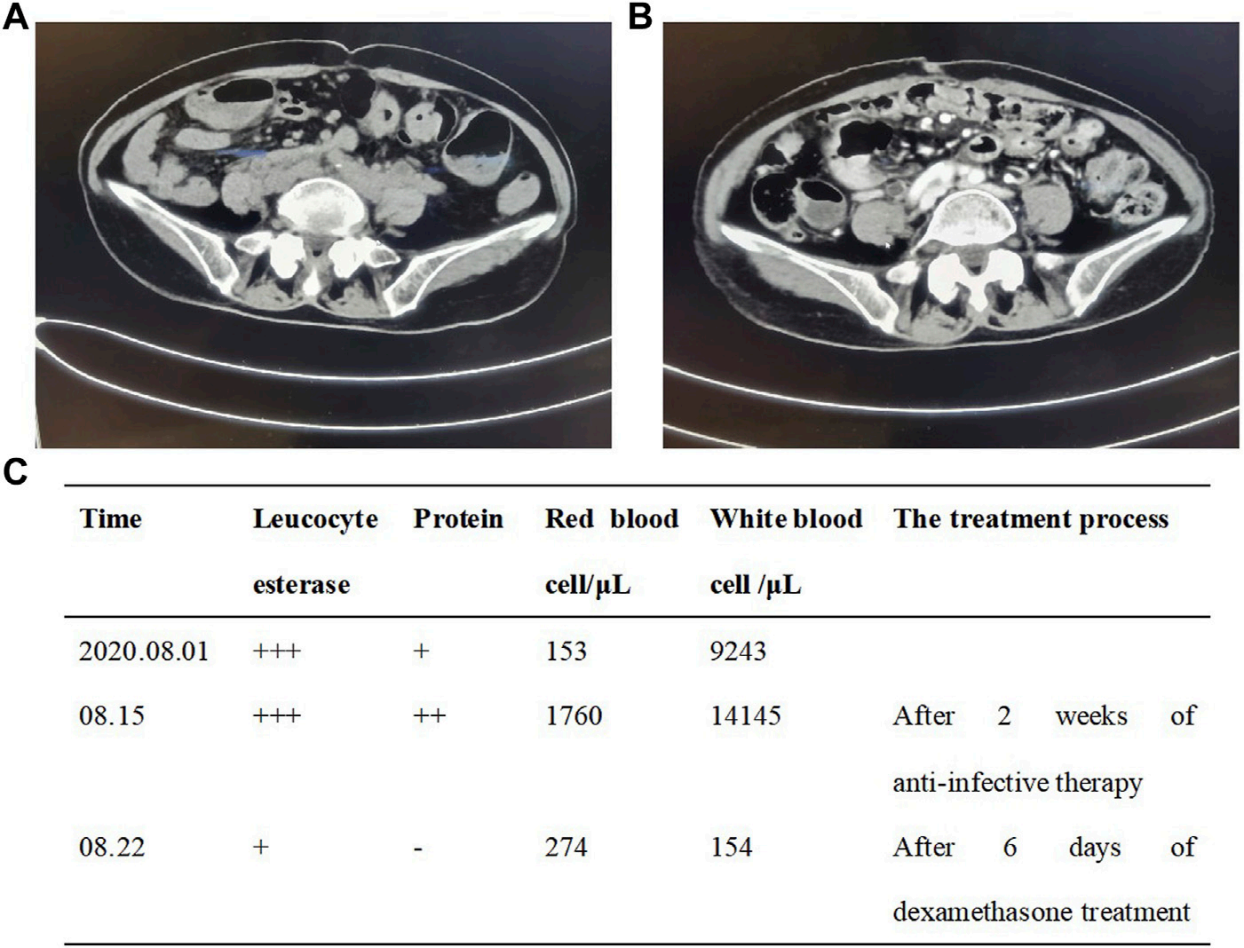
Pelvic CT imaging showed the patient’s immune ureteritis before **(A)** and after **(B)** the treatment of dexamethasone. The urine examination **(C)** of the patient.

A 80-year-old female was diagnosed with pancreatic cancer (cTxNxM1, Ⅳ, liver metastasis) in November 2019. Contrast-enhanced Magnetic Resonance Imaging (MRI) of the upper abdomen showed pancreatic head and uncinate process lesions and hepatic hemangioma in S2 and S3 segments (Figures 2A,C). Needle biopsy of pancreas revealed heterotypic cells, highly suspicious malignancy. The patient had a history of diabetes and was taking clonazepam as an antidepressant. On November 22, the patient underwent pancreatic HIFU, and was treated with gemcitabine (D1, D15, 1.2g, q4w). On December 16, sintilimab (a PD-1 monoclonal antibody) 200 mg was administered as the first-line treatment. On 15 January 2020, the patient underwent pancreatic HIFU. On January 16, February 5 and 22, gemcitabine (D1, 1g, q4w) plus sintilimab (D1, 200mg, q3w) were administered. Two months after sintilimab treatment, the patient developed severe diarrhea with >8 stools/d, weight loss of 2 kg and obvious abdominal pain. The patient was clinically diagnosed with immune colitis (CTCAE grade 3). The immune colitis recovered from the intravenous administration of methylprednisolone (2 mg/kg/d) for 5 days. The MRI imaging conferred significant tumor shrinkage (Figure 2B) and reduction of liver metastases on March 11(Figure 2D). The therapeutic evaluation of the patient was partial response (PR). On April 3 and 29, sintilimab plus gemcitabine were continued. Tumor markers significantly decreased (Figures 2E,F). Stable disease (SD) was evaluated. After treatment with sintilimab and gemcitabine on May 25, the patient was diagnosed with thrombocytopenia (CTCAE grade 2). Tebiol was given to raise platelets which led to a complete recovery within 5 days. After the treatment of sintilimab (D1, 200 mg) on July 31, the patient had frequent and urgent urination. Urine routine showed leucocyte esterase ++, protein +, red blood cell 153/μL and white blood cell 9243/μL. The infection of urinary tract was considered, but the patient had no fever, and repeated urine cultures of bacteria and fungi were negative. And pelvic CT of the patient was normal on May 21 during the earlier period of sintilimab treatment. On August 5, the patient’s depression worsened. After the consultation with the psychologist, mirtazapine was added to improve her depression. On August 14, pelvic CT (Figure 3A) showed that the wall of middle and lower sections of the left ureter was slightly thickened and dilated with fluid. Anti-infective treatments of ceftriaxone and levofloxacin were given for 2 weeks, and routine urine examinations showed no significant improvement. Thus the patient was diagnosed with PD-1 induced immune ureteritis. Sintilimab was discontinued and dexamethasone (5mg, qd, ivgtt) was given for 6 days. On August 22, routine urine examination markedly improved (Figure 3C). The patient’s urinary symptoms significantly relieved and CT imaging of urinary tract (Figure 3B) also showed improvement. The transurethral laser lithotripsy of ureteral/pelvis was performed on September 21. The patient’s second-line treatment regime was albumin paclitaxel (D1, 200 mg, Q3W) and HIFU. On November 6, enhanced CT of upper abdomen showed the tumor enlarged and liver metastases progressed. And tumor markers elevated (Figures 2E,F). The PFS of this patient was 11 months. On 5 January 2021, the patient’s third-line treatment regime was bevacizumab (D1, 200 mg) and oxaliplatin (D2, 100 mg). On March 11, enhanced CT of the upper abdomen showed that the pancreatic lesions enlarged and tumor progressed. CT of the pelvis showed no obvious abnormality. On March 17, the patient restarted immunotherapy. Bevacizumab (D0, 200 mg) + sintilimab (D1, 200 mg) + gemcitabine (D1, 1.0 g, Q2W) were given, and the patient’s vomiting reaction was severe. The patient’s treatment regime was adjusted to Gemcitabine (D1, 1 g, Q2W) and HIFU. In June, the patient developed anorexia, eating less, fatigue, and falling. The patient did not cooperate to take antidepressant drugs, and the medication adherence was poor. On July 6, the patient was treated with bevacizumab (D1, 100 mg) and erlotinib (150 mg) which led to a rash with pruritus that was intolerable. The patient died on 4 August 2021, and the main diagnosis of death was cachexia and depression. The OS of this patient has exceeded 20 months.

## 3 Discussion

We reported a case of a Chinese old female diagnosed with metastatic pancreatic cancer. After first-line treatment of Sintilimab plus gemcitabine combined with pancreatic HIFU, the patient achieved a remarkable benefit of 11-month PFS. Weiss GJ et al. indicated that gemcitabine plus pembrolizumab as the first-line treatment in pancreatic adenocarcinoma could achieve an improved PFS and OS of 8.1 months and 15 months, respectively (Weiss et al., 2018). In contrast, the combination of HIFU in the patient we reported resulted in the prolongation of PFS and OS by 3 and 5 months, respectively. A patient with metastatic pancreatic adenocarcinoma treated with FOLFIRINOX and gemcitabine plus nab-paclitaxel switched to irinotecan liposomal, at the same time was started on maintenance pembrolizumab and olaparib with no progression on CT surveillance for 8 months (Zhao et al., 2022). By contrast, the patient we reported achieved a remarkable benefit of 11-month PFS after first-line treatment of Sintilimab plus gemcitabine combined with pancreatic HIFU. To sum up, HIFU has a promising modality for the extension of survival time in advanced pancreatic patients. The ability of HIFU plus gemcitabine to control tumor outgrowth was moderately enhanced by adjuvant treatment with anti-PD-1 *via* adaptive immunity (Sheybani et al., 2020).

Two months after Sintilimab treatment, the patient occurred severe immune colitis. And the patient was diagnosed with immune ureteritis after 9 months of treatment. Chinese patients with advanced pancreatic cancer receiving immune therapy as a first-line treatment had prolonged survival compared with those receiving it as a second-line or multiple-line treatment, but the difference was not statistically significant. The immune-related adverse events that occurred were hypothyroidism, diarrhea, and rash (Sun et al., 2018). While the patient reported here presented immune colitis and rare immune ureteritis after PD-1 treatment. Chronic immune-mediated diarrhea can develop among patients with a more aggressive disease course and chronic features on colon histology. It likely reflects a prolonged immune checkpoint inhibitor effect and is associated with better cancer outcome and overall survival (Zou et al., 2020). Gastrointestinal-irAEs are associated with improved OS and PFS in patients with metastatic melanoma. Furthermore, higher grades of diarrhea are associated with even better patients’ OS rates (Abu-Sbeih et al., 2019). The patient we reported occurred immune colitis and rare immune ureteritis while also achieving a remarkable benefit of PFS and OS. The underlying mechanisms need to be further explored. It is worth noting that the elderly patient also suffered from depression. The combination treatment of Sintilimab, gemcitabine and HIFU for pancreatic cancer prolonged the patient’s OS, but the uncontrolled depression worsened the survival to rare and regrettable 20-month OS. Major depression is associated with worse survival in patients with common cancers (Walker et al., 2021). The association and clinical implications require further study.

This case demonstrated that PD-1 and HIFU are feasible and promising treatments for advanced pancreatic cancer. The first-line treatment of Sintilimab plus gemcitabine combined with HIFU demonstrates a potential therapeutic effect on metastatic pancreatic carcinoma with tolerable adverse reactions.

## Data Availability

The original contributions presented in the study are included in the article/supplementary material, further inquiries can be directed to the corresponding authors.
